# Predictors of Extubation Failure in Very Low Birth Weight Infants at a Tertiary Care Hospital in Al Ain: A Retrospective Study

**DOI:** 10.7759/cureus.55123

**Published:** 2024-02-28

**Authors:** Uzma Afzal, Nisha Varghese, Binu Pappachan, Zohra Siwji, Sameh Kasem, Nuralhuda Omar, Aiman Rahmani, Omar Abu Sa'da

**Affiliations:** 1 Pediatrics and Neonatology, Tawam Hospital, Al Ain, ARE

**Keywords:** neonatal intensive care unit, retrospective study, tertiary care hospital, very low birth weight infants, extubation failure

## Abstract

Objectives: To identify and analyze the factors leading to extubation failure among very low birth weight infants in a specific tertiary care setting in Al Ain, emphasizing clinical and demographic variables. The study used medical data of Very Low Birth Weight (VLBW) infants admitted to the Neonatal Intensive Care Unit (NICU) from 1st January 2015 to 31st December 2019, and evaluated the incidence and risk factors associated with extubation failure.

Methods: Data was collected from the hospital's electronic records and tabulated in Excel sheets, with extubation failure defined as reintubation due to deterioration of respiratory condition within seven days post-extubation. The data was collected from the period of 1st January 2015 to 31st December 2019. Inclusion criteria included babies admitted to the NICU with a gestational age of ≤ 32 weeks, or of birth weight ≤1500 grams who were intubated within the first seven days of life. Results were analyzed using SPSS software, version 9.0 (SPSS Inc., Chicago) to determine the risk factors for extubation failure and short-term outcomes.

Results: Gestational age, birth weight, antenatal steroids, mode of delivery, number of Survanta® (beractant intratracheal suspension) doses, Positive End-Expiratory Pressure (PEEP), Mean Airway Pressure (MAP), Mean Arterial Pressure (Blood Pressure (BP)), and Infectious Diseases (ID) (indicated by a positive blood culture) were found to be the key predictors of extubation failure in very low birth weight infants at a tertiary care hospital in Al Ain. The most common reasons for reintubation were FiO2 > 50% (23.53%), followed by Respiratory Acidosis (20.59%). Other factors, including maternal chorioamnionitis, Apgar scores, indication for intubation, caffeine, and pre-and post-extubation laboratory values, comorbidities, and hemoglobin (Hgb), creatinine and sodium levels were found to have no effect on the success of extubations.

Conclusions: The results of this research indicate that factors such as gestational age, birth weight, prenatal steroid use, delivery method, the quantity of Survanta® doses, PEEP, MAP, MAP (BP), and ID (+ve blood culture) were the primary determinants of unsuccessful extubation in VLBW babies at a tertiary healthcare facility in Al Ain. The predominant cause for needing reintubation was a FiO2 level above 50%, followed by Respiratory Acidosis. Additional ®®investigations are required to validate these findings and pinpoint other potential predictors of extubation failure within this demographic.

## Introduction

The respiratory system of very low birth weight (VLBW) infants is extremely fragile and vulnerable to complications when exposed to invasive mechanical ventilation (MV), such as pneumothorax, pneumonia, bronchopulmonary dysplasia (BPD), upper airway trauma, neurodevelopmental impairment, and death [1]. Reducing the duration of MV is, therefore, essential, with the primary objective being the reduction of these serious complications [1]. The extubation of VLBW infants, a critical step in this process, is complex and requires meticulous assessment due to the risk of complications associated with reintubation, including prolonged MV duration, increased hospital stay, and higher mortality rates [2-4]. Data contained records of VLBW infants admitted to the Neonatal Intensive Care Unit (NICU) from 1st January 2015 to 31st December 2019.

The significance and novelty of this study are derived from its focus on a comprehensive analysis of various predictors of extubation failure, a topic not thoroughly explored in previous research within this specific demographic and geographic context. The findings of this study are intended to contribute to the existing literature by providing insights into the unique risk factors for VLBW infants in Al Ain, thus aiding in the development of more effective extubation strategies. These strategies have the potential to enhance the success rate of extubation, thereby reducing the duration of MV and the length of hospital stays [2,5]. Ultimately, this could lead to a decrease in the risk of MV-associated complications and improve the long-term outcomes for VLBW infants [1,5].

## Materials and methods

This study was a retrospective observational study that analyzed the predictors of extubation failure in VLBW infants at Tawam Hospital, a tertiary care hospital in Al Ain. We focused on the medical records of all babies with a gestational age of ≤32 weeks or a birth weight of ≤1500 grams, admitted to the hospital's Neonatal Intensive Care Unit (NICU) from 1st January 2015 to 31st December 2019. Data were collected from the hospital's electronic records and tabulated in Excel sheets. The collected variables included demographics, ventilatory parameters, clinical condition, and blood gas analysis results prior to and post-extubation, as well as the mode of non-invasive ventilation setup post-extubation. Extubation failure was defined as re-intubation due to deterioration of respiratory condition within seven days post-extubation. The inclusion criteria for this study were babies admitted to the NICU with a gestational age of ≤ 32 weeks, or a birth weight of ≤1500 grams who were intubated within the first seven days of life. The exclusion criteria included infants identified to have chromosomal abnormalities, infants with critical congenital heart, lung, or gastrointestinal malformations, and infants who were not extubated from mechanical ventilation within four weeks of age. The data were analyzed using the Statistical Package for the Social Sciences (SPSS), version 9.0 (SPSS Inc., Chicago) to determine the risk factors for extubation failure and the short-term outcomes, including chronic lung disease and mortality.

For the study, data were collected on various variables pertaining to demographics, ventilatory parameters, clinical condition, and blood gas analysis results prior to and post-extubation and before re-intubation, as well as the mode of non-invasive ventilation setup post-extubation. These variables included the sex of the infant, birth weight in grams, gestational age in weeks, administration of antenatal steroids, maternal chorioamnionitis, mode of delivery, Apgar score, surfactant usage, age of first intubation, reason for intubation, caffeine administration, caffeine maintenance dose, Positive Inspiratory Pressure (PIP), Positive End-Expiratory Pressure (PEEP), Fraction of Inspired Oxygen (FiO2), Mean Airway Pressure (MAP), pH, Partial Pressure of Carbon Dioxide (PCO2), Bicarbonate (HCO3), lactate, Mean Arterial Pressure (MAP, Blood Pressure), Cardiovascular System (CVS) comorbidities, hemodynamic significance of Patent Ductus Arteriosus (PDA), ionotropes, hemoglobin level, metabolic comorbidities such as creatinine level and sodium level, Gastrointestinal (GI) comorbidities, Infectious Diseases (indicated by a positive blood culture), type of Non-Invasive Ventilation (NIV), biphasic Continuous Positive Airway Pressure (CPAP), and need for reintubation within seven days. The criteria for extubation at our unit during the study period were a Fraction of Inspired Oxygen (FiO2) of 30%, Mean Airway Pressure (MAP) between 8-9, and blood gas parameters including a pH greater than 7.25 and a Partial Pressure of Carbon Dioxide (PCO2) less than 55. Post extubation, the mode of respiratory support was predominantly Non-invasive Ventilation (NIV) for infants who were successfully extubated, while those who failed typically received Assisted Control Ventilation (ACV) with Volume Guarantee (VG).

The statistical analysis involved using Student's t-test for continuous variables, and Chi-square or Fisher's exact test for categorical variables. Binary logistic regression analysis was performed to determine the main risk factors for extubation failure. Additionally, Python's pandas and NumPy libraries were utilized for data manipulation and analysis. Descriptive statistics, including frequencies and percentages for categorical data and means and standard deviations for continuous data, were employed. Tables were used to display the results of the analysis. Hypothesis testing methods such as t-tests, Chi-square tests, and ANOVA were utilized to compare differences between groups and to find the p-value in the analysis.

## Results

Study population and demographics

In this retrospective analysis, we evaluated 61 very low birth weight infants at a tertiary care hospital in Al Ain, categorized based on extubation outcomes into two groups: extubation failure (34 infants) and extubation success (27 infants). Gender distribution showed no significant difference in extubation outcomes, with 73.53% males in the failure group and 55.56% in the success group (p=0.23).

Clinical outcomes and measurements

Birth Weight and Gestational Age

**Figure 1:**
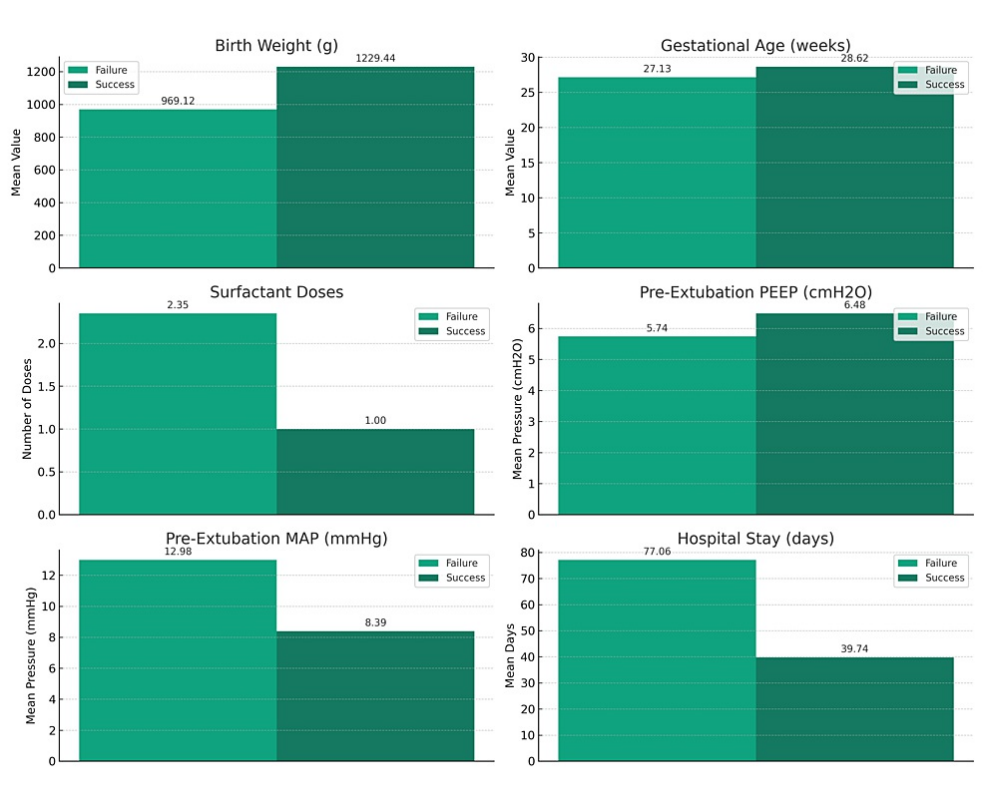
Significant factors by extubation outcome

The infants who experienced extubation failure had a significantly lower mean birth weight (969.12 ± 215.30 g) compared to those in the success group (1229.44 ± 248.34 g, p=0.01). Similarly, the mean gestational age was lower in the failure group (27.13 ± 1.60 weeks) versus the success group (28.62 ± 1.84 weeks, p=0.01), indicating that both lower birth weight and gestational age are critical factors in extubation outcomes. These significant disparities are visually represented in Figure 1.

Pre-Extubation Parameters

Our findings indicated no significant differences in maternal chorioamnionitis, mode of delivery, and Apgar scores between the groups. However, surfactant administration emerged as a significant factor; infants who received only one dose of surfactant were more likely to succeed in extubation compared to those who received multiple doses (p=0.03). This significant relationship is further detailed in Table 1, presenting demographic characteristics and clinical parameters.

**Table 1:** Variable descriptive analysis Apgar1, Apgar5, Apgar10 - Apgar Score at 1 minute, 5 minutes, and 10 minutes after birth respectively; Ant. Steroid - Antenatal Steroid; BP - Blood Pressure; BW - Body Weight; CPAP - Continuous Positive Airway Pressure; Cr - Creatinine; CVS Comorb - Cardiovascular Comorbidity; FiO2 - Fraction of inspired oxygen; GA - Gestational Age; GI Comorbid - Gastrointestinal Comorbidity; HCO3 - Bicarbonate; Hgb - Hemoglobin; Hosp - Hospital; ID (+ve Bld Cx) - Infectious Diseases (Positive Blood Culture); LSCS - Lower Segment Caesarean Section; MAP - Mean Airway Pressure; NCPAP - Nasal Continuous Positive Airway Pressure; NEC - Necrotizing Enterocolitis; NIV - Non-Invasive Ventilation; NIV PC - Non-Invasive; Ventilation Pressure Control; Na - Sodium; PDA - Patent Ductus Arteriosus; PC - Pressure Control; PEEP - Positive End-Expiratory Pressure; PCO2 - Partial Pressure of Carbon Dioxide; PIP - Peak Inspiratory Pressure; Rx - Prescription / Treatment; SVD - Spontaneous Vaginal Delivery; Survanta® - beractant intratracheal suspension).

Characteristics	Extubation Failure ( N = 34)	Extubation Success (N = 27)	P Value
Sex, count (%)			0.23
Male	25 (73.53%)	15 (55.56%)	
Female	9 (26.47%)	12 (44.44%)	
TOTAL	34 (100%)	27(100%)	
BW, in grams, Mean (Std. Dev)	969.12 (215.30)	1229.44 (248.34)	0.01
GA, in weeks, Mean (Std. Dev)	27.13 (1.60)	28.62 (1.84)	0.01
Ant. Steroid, count (%)			0.24
2 Doses	18 (52.94%)	17 (62.96%)	
1 Dose	8 (23.53%)	8 (29.63%)	
0 Doses	8 (23.53%)	2 (7.41%)	
Maternal Chorioamnionitis (on Rx), count (%)			0.63
No	29 (85.29%)	25 (92.59%)	
Yes	5 (14.71%)	2 (7.41%)	
Mode of delivery, count (%)			0.24
LSCS	25 (73.53%)	24 (88.89%)	
SVD	9 (26.47%)	3 (11.11%)	
Apgar Score, count (%)			
Apgar1	5.26 (1.69)	5.52 (2.12)	0.6
Apgar5	7.06 (1.30)	7.67 (1.14)	0.06
Apgar10	8.18 (1.00)	8.56 (1.01)	0.15
Survanta ® , count (%)			0.03
1 Dose	6 (17.65%)	14 (51.85%)	
2 Doses	12 (35.29%)	5 (18.52%)	
3 Doses	11 (32.35%)	4 (14.81%)	
4 Doses	5 (14.71%)	4 (14.81%)	
Age of 1st intubation	1.38 (1.61)	1.07 (0.38)	0.34
Reason (indication) for intubation, count (%)			0.77
FiO2 > 30%	17 (50.00%)	13 (48.15%)	
Recurrent apnea	13 (38.24%)	12 (44.44%)	
pH<7.30, PCO2 >60	2 (5.88%)	2 (7.41%)	
FiO2 > 30%, pH<7.30, PCO2 >60	1 (2.94%)	0(0%)	
Recurrent apnea, FiO2 > 30%	1 (2.94%)	0(0%)	
Caffeine, count (%)			0.84
Yes	33 (97.06%)	25 (92.59%)	
No	1 (2.94%)	2 (7.41%)	
Caffeine maintenance dose, count(%)			1.00
10mg/Kg	27 (79.41%)	21 (77.78%)	
5mg/Kg	7 (20.59%)	6 (22.22%)	
BEFORE EXTUBATION			
PIP, in cmH2O, Mean (Std. Dev)	17.18 (3.71)	18.41 (2.86)	0.16
PEEP, in cmH2O, Mean (Std. Dev)	5.74 (0.67)	6.48 (0.98)	0.013
FiO2, in %, Mean (Std. Dev)	24.76 (4.02)	24.93 (5.87)	0.9
MAP, in mmH, Mean (Std. Dev)	12.98 (9.84)	8.39 (1.43)	0.02
pH, Mean (Std. Dev)	7.31 (0.07)	7.32 (0.06)	0.23
PCO2, in mmHg, Mean (Std. Dev)	42.53 (9.04)	42.81 (7.29)	0.9
HCO3, in mEq/L, Mean (Std. Dev)	20.86 (2.55)	22.17 (3.42)	0.09
Lactate, in mmol/L, Mean (Std. Dev)	2.79 (1.43)	2.29 (1.30)	0.16
MAP (BP), in mmHg, Mean (Std. Dev)	36.71 (5.98)	40.22 (6.58)	0.03
CVS Comorb, count(%)			0.34
NIL	21 (61.76%)	20 (74.07%)	
Hemodynamic sig. PDA	7 (20.59%)	6 (22.22%)	
Ionotropes	3 (8.82%)	1 (3.70%)	
Ionotropes, Hemodynamic sig. PDA	3(8.82%)	0(0%)	
Hgb level, g/dL, Mean (Std. Dev)	14.20 (2.51)	15.57 (3.22)	0.07
Metabolic Comorb- Cr level, mg/d, Mean (Std. Dev)	78.06 (28.97)	76.78 (33.70)	0.87
Na level, mEq/L, Mean (Std. Dev)	138.68 (5.69)	138.70 (4.83)	0.98
GI Comorbid., count(%)			0.21
Nil	28 (82.35%)	26 (96.30%)	
NEC Stage 3	4 (11.76%)	1 (3.70%)	
NEC Stage 2	2 (5.88%)	0(0%)	
ID (+ve Bld Cx), count(%)			0.021
No	20 (58.82%)	26 (96.30%)	
Yes	14 (41.18%)	1 (3.70%)	
POST EXTUBATION			
Type of NIV, count(%)			0.46
NIV PC	29 (85.29%)	20 (74.07%)	
NCPAP	3 (8.82%)	3 (11.11%)	
Biphasic CPAP	2 (5.88%)	4 (14.81%)	
PIP, in cmH2O, Mean (Std. Dev)	17.09 (5.18)	18.59 (3.74)	0.21
PEEP, in cmH2O, Mean (Std. Dev)	6.76 (1.46)	6.89 (1.12)	0.72
FiO2, in %, Mean (Std. Dev)	27.29 (4.85)	24.67 (6.53)	0.08
OUTCOME			
Mortality, count(%)			0.16
No	28 (82.35%)	17 (62.96%)	
Yes	6 (17.65%)	10 (37.04%)	
Length of Hosp. stay, in Days, Mean (Std. Dev)	77.06 (40.62)	39.74 (31.26)	0.02

Respiratory Support and Blood Gas Levels

Before extubation, the failure group exhibited higher levels of PEEP and MAP, which were statistically significant (p=0.02 for both). This suggests that higher requirements for respiratory support before extubation could predict extubation failure. Conversely, blood gas parameters such as pH, PCO2, HCO3, and lactate did not significantly affect extubation outcomes. These results are elucidated in Figure 1.

Comorbidities and Post-Extubation Support

The incidence of cardiovascular, metabolic, gastrointestinal comorbidities, and infectious diseases showed no significant correlation with extubation success. The type of non-invasive ventilation post-extubation, such as NIV PC, did not significantly differ between the groups, suggesting that these factors may not be as critical in predicting extubation outcomes.

Outcomes Post-Reintubation

Following reintubation, the majority of infants were on NIV PC, highlighting the commonality of this support mode. The primary reasons for reintubation included high FiO2 requirements (>50%) and respiratory acidosis, indicating severe respiratory challenges. Interestingly, 91.18% of reintubated infants had negative blood cultures, which contrasts with the initial assumption that infection status might influence reintubation rates. Complications post-reintubation, such as necrotizing enterocolitis (NEC) and intraventricular hemorrhage (IVH) were observed, underscoring the vulnerability of this population to serious health issues following failed extubation attempts. A detailed breakdown of these complications and their frequency is provided in Table 2.

**Table 2:** Variables analysis for babies who failed extubation before reintubation CLD - Chronic Lung Disease; CPAP - Continuous Positive Airway Pressure; Cx - Culture; FiO2 - Fraction of Inspired Oxygen; Gr - Grade; HCO3 - Bicarbonate; HFNC - High Flow Nasal Cannula; HUS - Hyperechoic Ultrasound; IVH - Intraventricular Hemorrhage; mEq/L - milliequivalents per Liter; mmHg - millimeters of Mercury; mmol/L - millimoles per Liter; Mod - Moderate; Na - Sodium; NEC - Necrotizing Enterocolitis; NIL - Not Indicated/Listed; NIV - Non-Invasive Ventilation; PC - Pressure Control; PEEP - Positive End-Expiratory Pressure; PCO2 - Partial Pressure of Carbon Dioxide; PIP - Peak Inspiratory Pressure; Resp. - Respiratory; ROP - Retinopathy of Prematurity; Std. Dev - Standard Deviation; cmH2O - centimeters of Water; wks - weeks.

Type of NIV, count(%)	Results
NIV PC	29 (85.29%)
HFNC	3 (8.82%)
Biphasic CPAP	2 (5.88%)
PIP, in cmH2O, Mean(Std. Dev)	19.18 (5.25)
PEEP, in cmH2O, Mean(Std. Dev)	7.38 (1.13)
FiO2, in %, Mean(Std. Dev)	36.03 (11.79)
pH, Mean(Std. Dev)	7.21 (0.09)
PCO2, in mmHg, Mean(Std. Dev)	55.36 (14.75)
HCO3, in mEq/L, Mean(Std. Dev)	20.84 (3.25)
Lactate, in mmol/L, Mean(Std. Dev)	2.74 (1.77)
Na level, mEq/L, Mean(Std. Dev)	140.18 (5.69)
Reason re-intubation, count(%)	
FiO2 > 50%	8 (23.53%)
Resp. Acidosis	7 (20.59%)
Mod‐Severe Resp. Distress	7 (20.59%)
Recurrent Apnea	7 (20.59%)
Mod‐Severe Resp. Distress, Recurrent Apnea	2 (5.88%)
Mod‐Severe Resp. Distress, Resp. Acidosis	1 (2.94%)
Mod‐Severe Resp. Distress,Resp. Acidosis, Recurrent Apnea	1 (2.94%)
Other	1 (2.94%)
Risk factor for reintubation, count(%)	
Negative Blood Cx	31 (91.18%)
Positive Blood Cx	3 (8.82%)
Total Days on ventilator, day, Mean(Std. Dev)	11.18 (9.05)
CLD (Need for any resp support after 36wks), count(%)	
Yes	20 (58.82%)
No	14 (41.18%)
ROP, count(%)	
No	31 (91.18%)
Yes	3 (8.82%)
NEC (post reintubation), count(%)	
NIL	25 (73.53%)
Stage 3	5 (14.71%)
Stage 2	4 (11.76%)
IVH (Brain HUS post re-intubation), count(%)	
NIL	22 (64.71%)
IVH Gr 1	6 (17.65%)
IVH Gr 2	4 (11.76%)
IVH Gr 3	1 (2.94%)
IVH Gr 4	1 (2.94%)

The analysis provided a clear delineation between factors influencing extubation outcomes. Birth weight, gestational age, and pre-extubation respiratory support parameters were significantly associated with failure, highlighting their importance in clinical assessment and decision-making. Moreover, the length of hospital stay significantly differed between the groups, reinforcing the impact of successful extubation on hospitalization duration (p=0.02). This important finding is further illustrated in Figure 1, which aggregates these significant factors into a comprehensive visual format.

## Discussion

Descriptive statistics were used to analyze the data, forming the basis for the conclusions made in this study. The results of the analysis showed that gestational age, birth weight, mode of delivery, number of Survanta® (beractant intratracheal suspension) doses, PEEP, MAP, MAP (BP), and ID (+ve blood culture) were found to be the key predictors of extubation failure in very low birth weight infants at a tertiary care hospital in Al Ain. The most common reasons for reintubation were FiO2 > 50% (23.53%), followed by Respiratory Acidosis (20.59%)

The findings of this study are in agreement with the literature, which shows that GA, birth weight, antenatal steroids, and gestational age are significant predictors of extubation failure among neonates [1-3]. Similarly, the results of this study show that higher birth weights are associated with successful extubation (p=0.00), while lower birth weights are associated with extubation failure (p=0.23). Additionally, the findings of the study are not in accord with the literature, which suggests that premature neonates receiving antenatal steroids before delivery are more likely to survive than those who do not, and thus have a higher chance of successful extubation [4,5].

The results of this study found that the mode of delivery has no statistically significant impact on the success of extubation in very low birth weight infants (p=0.24). This finding contrasts with some literature, which has suggested that caesarean section delivery is associated with increased mortality and lower chances of extubation success compared to normal vaginal delivery [6,7]. Additionally, the results of this study show that Survanta® is a key predictor of extubation failure, with lower chances of successful extubation among neonates receiving only one dose of Survanta® (p=0.03). This is in agreement with the literature, which suggests that lower doses of Survanta® are associated with poorer ventilatory management, which can lead to extubation failure [8].

The findings of this study also demonstrate that MAP (BP) is a key predictor of extubation failure in very low birth weight infants (p=0.03), with higher risks of failure associated with lower MAP (BP). This is in line with the literature, which suggests that lower blood pressure is associated with poorer responses to extubation, leading to increased chances of failure [9,10]. Additionally, the results of this study show that the type of NIV is not an important predictor of success or failure of extubation (p=0.46). This is in agreement with the literature, which suggests that post-extubation use of NIV and intermittent positive pressure ventilation (IPPV) as preparatory approaches for extubation improves the chances of successful extubation and decreases the risk of failure [11,12].

In terms of PEEP levels, the mean PEEP before reintubation was 7.38, which is slightly below the international standard of 8-10 cm H2O. Low PEEP may be a risk factor for reintubation, as it may lead to a decrease in lung compliance, resulting in an increase in airway resistance and alveolar collapse. Similarly, the mean FiO2 before reintubation was 36.03, which is above the international standard of 21-30%. High FiO2 levels may indicate the development of respiratory failure and an increased risk of reintubation. The mean pH before reintubation was 7.21, indicating a slight alkalosis. Alkalosis is usually caused by hyperventilation and may lead to a decrease in oxygenation and an increased risk of reintubation. The mean PCO2 before reintubation was 55.36, which is slightly higher than the normal range of 35-45 mmHg. Elevated PCO2 levels may be a sign of respiratory acidosis, which is one of the most common reasons for reintubation in very low birth weight infants. The mean HCO3 before reintubation was 20.84, which is within the normal range of 18-25 mmol/L. The mean lactate before reintubation was 2.74, which is slightly higher than the normal range of 0.5-2.0 mmol/L. Elevated lactate levels may indicate an increased risk of reintubation. The mean sodium level before reintubation was 140.18, which is within the normal range of 135-145 mmol/L.

It should be noted that there are some limitations to this study. The study was retrospective and only a small sample size was used, so it is possible that the conclusions drawn may not be applicable to the broader population. Additionally, the results from this study can only be applied to the context of this particular hospital in Al Ain, UAE. Furthermore, due to the retrospective nature of the study, there may be some discrepancies between the actual and recorded variables.

## Conclusions

In conclusion, our study advances the understanding of the complex interplay between various factors and extubation failure in VLBW infants, within the context of Al Ain's tertiary care environment. The results of this study suggest that FiO2 > 50%, respiratory acidosis, moderate-severe respiratory distress, and recurrent apnea are the most common reasons for reintubation in this population. The findings of this study suggest that gestational age, birth weight, antenatal steroids, mode of delivery, Number of Survanta® doses, PEEP, MAP, MAP (BP), and ID (+ve blood culture) were the key predictors of extubation failure in very low birth weight infants at a tertiary care hospital in Al Ain. The most common reason for reintubation was FiO2>50%, followed by Respiratory Acidosis. Further studies are needed to confirm these results and to identify other predictors of extubation failure in this population. Furthermore, the results indicate that NEC and intraventricular hemorrhage IVH are common post-reintubation complications in this population. The results of this study will help inform clinicians in the management of VLBW infants in the NICU and provide valuable insight into the factors that may predict extubation failure and post-reintubation complications.
